# Detection of diphtheria toxin production by toxigenic corynebacteria using an optimized Elek test

**DOI:** 10.1007/s15010-022-01903-x

**Published:** 2022-09-02

**Authors:** Vyacheslav G. Melnikov, Anja Berger, Andreas Sing

**Affiliations:** 1 National Conciliary Laboratory on Diphtheria, Veterinärstraße 2, 85764 Oberschleißheim, Germany; 2 Department of Public Health Microbiology, Bavarian Health and Food Safety Authority (LGL), Veterinärstraße 2, 85764 Oberschleißheim, Germany

**Keywords:** Elek test, *Corynebacterium ulcerans*, Diphtheria toxin detection, Diphtheria, Laboratory diagnosis

## Abstract

**Purpose:**

Diphtheria, still present in many countries of the world, is caused by toxigenic strains of species of the *Corynebacterium diphtheriae* complex, mainly *Corynebacterium diphtheriae* and the emerging zoonotic pathogen *C. ulcerans*. The immunoprecipitation test according to Elek is the gold standard for detection of the major virulence factor diphtheria toxin (DT) in toxigenic corynebacteria. Due to its sophisticated methodological requirements, the classical Elek test is performed mainly by specialized reference laboratories. It was revealed that the current modification of the Elek test does not detect the toxin in weakly toxigenic isolates. Therefore, a more robust method for detecting free DT is urgently needed, especially for toxigenic *C. ulcerans* strains which are known to produce often much lower amounts of DT than *C. diphtheriae*.

**Methods:**

Thirty-one *tox*-positive *C. ulcerans* isolates with a negative standard Elek test result previously determined as NTTB (non-toxigenic *tox* bearing) were re-analyzed in this study using a modified immunoprecipitation method optimized regarding different parameters including type and concentration of antitoxin, medium volume, inoculum distance from the antitoxin disk and position of controls.

**Results:**

All 31 *C. ulcerans* strains tested positive in the optimized Elek test.

**Conclusion:**

Only with a reliable and easy-to-handle method for detecting the toxigenicity of *C. ulcerans*, it is possible to assess the etiological role of this emerging zoonotic bacterium in human pathology.

## Introduction

Diphtheria toxin (DT) producing strains of the classical diphtheria agent *Corynebacterium diphtheriae* and the emerging zoonotic species *Corynebacterium ulcerans* are the most important pathogens of the *C. diphtheriae* complex causing diphtheria and diphtheria-like illness in humans. Since the phage-encoded DT is the virulence factor responsible for causing diphtheria symptoms, i.e., pseudomembrane formation or cardiac and neurological sequelae, only toxigenic strains can cause classical respiratory or cutaneous diphtheria, while non-toxigenic strains are not able to do so. Therefore, identification of toxigenic *Corynebacterium* strains is of utmost importance in diphtheria laboratory-based diagnosis, both for managing the individual patient as well as for public health measures. Detection of the DT encoding *tox* gene can nowadays easily be achieved by PCR. *Tox* positivity by PCR, however, does not always match with toxin production, because non-toxigenic strains of *C. diphtheriae* bearing a non-functional toxin gene (NTTB) are circulating worldwide [1, 2].

Elek test is the gold standard for detection of the DT. This immunoprecipitation assay, also known as Elek-Ouchterlony assay, is a reaction between an antigen (DT) and an antibody (antitoxin) with the formation of a visible precipitate. This method, described in 1949 by Elek [3] has replaced the in vivo diphtheria virulence test in guinea pigs or rabbits. A modification of the Elek test was proposed by the Ukrainian scientists Feldman et al. [4]. This method differs by the use of antitoxin-impregnated paper discs around which bacterial cultures were inoculated in rounded plaques. This Elek test modification was combined with a purified diphtheria antitoxin preparation and the dried nutrient medium developed and commercially produced in Russia [4]. This method has proven itself well in assessing the toxin-expressing capacity of *C. diphtheriae* strains in times of diphtheria epidemics in Russia, Ukraine and other former USSR countries in the 1990s [5]. Based on the above method, Engler et al. [6] modified the Elek test; this version became applicable for DT detection throughout the world and was also included in the very recently published WHO Laboratory Manual for the diagnosis of diphtheria and other related infections [6–9].

Among the strains deposited in the German Conciliary Laboratory on Diphtheria (GCLoD) culture collection, 31 *tox* gene-positive isolates of *C. ulcerans* were found, which were negative in the Elek test and therefore classified as NTTB. At the same time, all these isolates appeared positive in a Lateral Flow Immunoassay (LFIA), sensitive and specific test recently designed for reliable DT detection [10]. It has been supposed that current Elek test does not disclose the toxin in weakly toxigenic isolates. We have made some changes to the immunoprecipitation method, and here we provide a protocol for the optimized Elek test with the capacity to detect toxigenic corynebacteria, including those with low toxin production.

## Materials and methods

Thirty-one *C. ulcerans* isolates from the GCLoD bacterial strain collection were used. They were both of human and animal origin and isolated in Germany in 2011–2022. The presence of the toxin gene in these isolates was determined by PCR [8]. DT was determined by the Elek test [6]. The *tox* gene-positive, Elek-negative strains were named KL 297, KL 345, KL 367, KL 381, KL 392, KL 442, KL 475, KL 492, KL 497, KL 501, KL 515, KL 540, KL 547, KL 556, KL 568, KL 846, KL 860, KL 900, KL 1294, KL 1357, KL 1363, KL 1370, KL 1779, KL 1809, KL 1825, KL 1865, KL 1934, KL 1972, KL 1986a, KL 1986b, KL 1989. *C. diphtheriae* NCTC 3984 (weakly toxigenic *C. diphtheriae* subsp. *gravis*) and NCTC 10356 (non-toxigenic *tox* gene-negative *C. diphtheriae* subsp. *belfanti*) were used for the optimized Elek test as a positive and a negative control strain, respectively. Cultures for PCR and Elek test were grown on Columbia Blood agar (Oxoid) and Hoyle Tellurite Agar (Becton Dickinson) for 24 h.

### Elek test

**Fig. 1:**
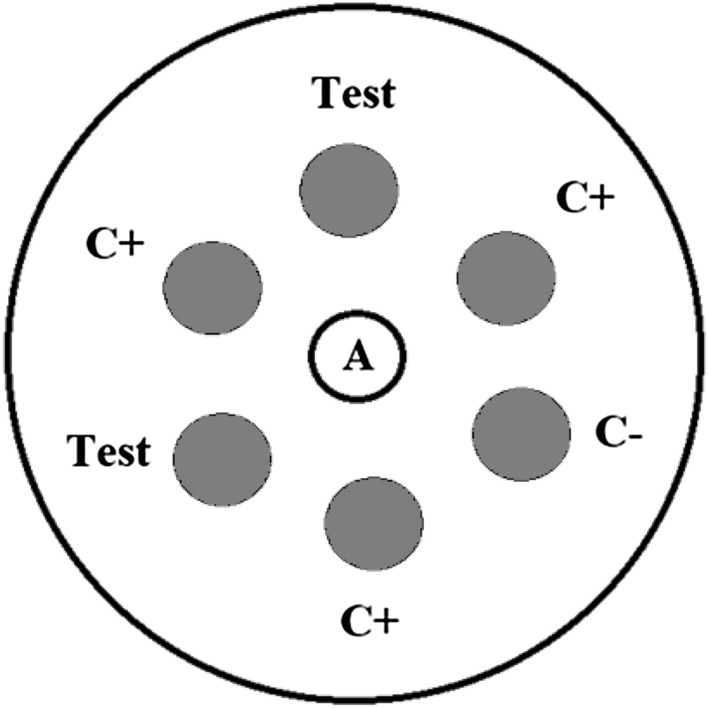
Design of the optimized Elek test. A—Disc with antitoxin; Test—culture tested; C+ positive control strain; C− negative control strain

**Fig. 2:**
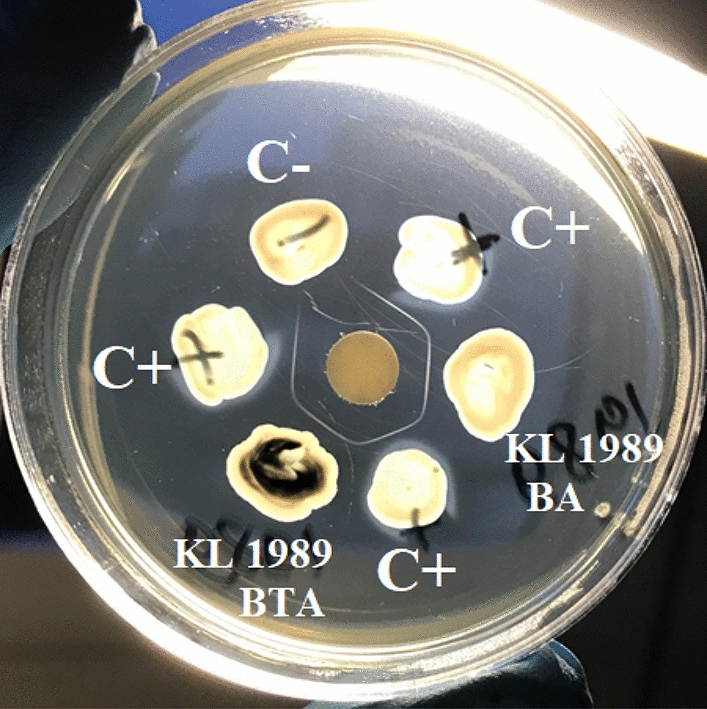
Optimized Elek test with KL 1989 testing isolate after 24 h of incubation at 37 °C with non-purified antitoxin. BA—test culture from Blood Agar; BTA—test culture from Blood Tellurite Agar. Specific precipitin lines are seen against positive controls and test cultures, but not against negative control

The Elek agar base was prepared as described previously [6, 8]. A molten and cooled to 50 °C Elek agar (4 ml) was supplemented with 1 ml (20%) of Bovine Calf Serum (Sigma-Aldrich, Taufkirchen, Germany), mixed gently and poured into 5.5 cm diameter Petri dish (Sarstedt, Nümbrecht, Germany). The medium was spread over the bottom by gently swirling the plate. Then the plate was left in a laminar flow hood for an hour with the ajar lid to solidify the medium and dry its surface. Such plates can be stored for 3–5 days in refrigerator at 4 °C. 20 μl of purified diagnostic diphtheria antitoxin (Microgen, Moscow, Russia) at a concentration of 125 μl/ml in a sterile distillate water or non-purified antitoxin for treatment of diphtheria (Microgen) at a concentration 250 μl/ml was applied to a 6 mm paper disk (Whatman GE Healthcare, Maidstone, UK), dried in a laminar flow hood for 2 h and stored at + 4 °C in a sealed container. A disk with antitoxin was placed on the agar and 6 microbial plaques were inoculated around the antitoxin disc agar using disposable plastic loops with a capacity of 1 μl: 3 plaques of the toxigenic control strain, 1 plaque of the non-toxigenic control strain, and two plaques of the same test strain (Fig. 1). The result of the reaction was regarded as positive if a precipitin line was formed between the bacterial plaque and the disk loaded with antitoxin after 24 h of Elek test incubation at 37 °C. The precipitin line should merge with the lines formed by the plaques of the control strain located on both sides of the test strain (Fig. 2). All experiments were carried out in triplicate.

## Results

A comparison of two nutrient media for Elek test: (a) Korinetoksagar (SCRAMB, Obolensk, Russia), commercially available dried medium and (b) in-house-made medium [8], demonstrated an advantage of the WHO Manual’s recommended one (data not shown), therefore, it was used in our study. The concentration of the antitoxin, thickness (volume) of the medium, density of the culture and distance of inoculum from the antitoxin disc were also optimized in preliminary experiments (data not shown). Immunoprecipitation with clear bands occurred more rapidly when a volume of the medium of 5 ml, a concentration of the antitoxin of 2.5 IU per disc and a heavy inoculum (full loop) were used. The optimal distance between the inoculum and the antitoxin disc was found to be 6 mm. Diameter of the bacterial plaques were also 6 mm. We noticed that the precipitin lines of the test culture were better expressed if this plaque was placed in between the plaques of the control toxigenic strain. In this regard, we have proposed the Elek test format indicated on Fig. 1. It is worth mentioning that toxigenic *C. diphtheriae* (including toxigenic control strain NCTC 3984) in the optimized version of the Elek test produce precipitin lines earlier—in 16–18 h.

**Fig. 3:**
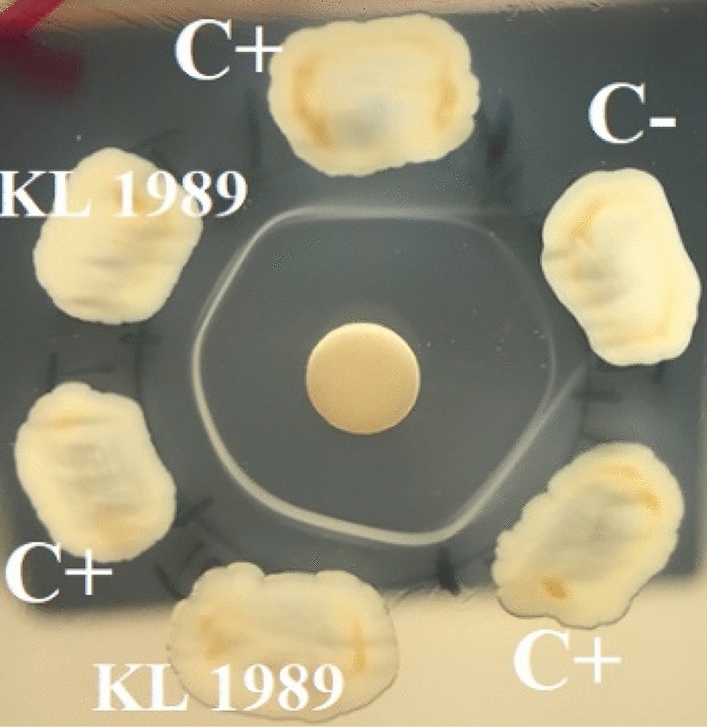
Optimized Elek test with KL 1989 testing isolate after 48 h of incubation at 37 °C with non-purified antitoxin. Non-specific precipitin line is seen against negative control

The purity of the antitoxin is important for the Elek test. Non-purified therapeutic diphtheria antitoxin preparations are produced from the blood of equines immunized with diphtheria vaccine and possess antibodies also to proteins other than DT. Such proteins are derived from the *C. diphtheriae* PW8 vaccine strain cells [11]. If the antitoxin is non-purified, non-specific lines of immunoprecipitation after prolonged incubation of the Elek test (48 h) can be seen (Fig. 3). Such precipitin lines in non-toxigenic strains are often misinterpreted as a positive result. Diphtheria antitoxin, purified by specific absorption and containing only antibodies against DT, is more preferable for the Elek test. If the purified antitoxin is not available, the non-purified therapeutic antitoxin could be used with the optimized version of the Elek test if the result is read at 24 h, since the non-specific precipitin lines appear only after 48 h.

In our experiments, we found that all 31 *C. ulcerans* isolates studied were DT positive in the optimized Elek test both with purified and non-purified antitoxin. It was also demonstrated that the bacterial culture for the Elek test could be taken both from Blood Agar and Hoyle Tellurite Agar (Fig. 2). Therefore, for toxigenicity testing, it is possible to use not only isolated and purified cultures of corynebacteria, but also suspicious black colonies grown on the primary inoculation tellurite plate.

## Discussion

In the past, the assessment of the toxigenicity of *C. ulcerans* has not played an important role, since *C. diphtheriae* was considered the only causative agent of diphtheria [4, 5, 9, 12]. However, within the last several years, toxigenic *C. ulcerans* was recognized as an emerging zoonotic pathogen causing diphtheria and diphtheria-like illness even outnumbering infections caused by toxigenic *C. diphtheriae* in many industrialized countries [12–14]. Thus, the detection of DT in *C. ulcerans* (often weakly toxigenic) becomes a very important task for interpreting the etiological role of this pathogen. As shown by external quality assessments of the European Diphtheria Surveillance Network (EDSN), most of the laboratories participating in the study had difficulty testing low-toxigenic strains of *C. ulcerans* using the Elek method [15].

In the present study, the advantages and disadvantages of previous Elek test modifications were considered and the method was improved for detecting pathogenic corynebacteria, including weakly toxigenic strains. The most critical point was found to be the distance of the bacterial inoculum from the antitoxin disc. The optimal spacing was 6 mm, similar to that described by Feldman et al. [4], despite the fact that the authors did not test isolates of *C. ulcerans*. Engler et al. [6] suggested a spacing of 9 mm, probably because they did not test low-toxigenic *C. ulcerans* isolates. Also, Engler et al. [6] used rectangular rather than round plaques. The round shape of the bacterial inoculum and a spacing of 6 mm appears to create an optimal quantitative ratio between toxin and antitoxin. In addition, the round shape of the plaques reduces the possibility of cross-contamination between adjacent plaques. The specificity of the test could be increased due to the use of a purified antitoxin, which does not produce false positive precipitin lines. When using purified antitoxin, the result of the Elek test can be evaluated not only after 24 h, but even after 48 h. After 48 h of Elek test cultivation, the precipitin lines become much more pronounced. If non-purified antitoxin is used here, the result can only be determined after 24 h, but cannot not be read later due to the possibility of non-specific precipitation (Fig. 3). The Elek test has also been simplified—now not two, but only one positive control is used, and the amount of antitoxin has been reduced.

The very recent WHO guidelines [8] recommend the following methodology for the laboratory diagnosis of diphtheria: isolation of a pure culture, biochemical identification as possible *C. diphtheriae* or *C. ulcerans*, and finally testing cultures for their ability to produce DT, which takes 5–6 days. In our study it was demonstrated that the bacterial culture for the Elek test could be taken not only from Blood Agar but also from Hoyle Tellurite Agar. So, a different diphtheria diagnostic approach could be proposed—direct application of suspicious black colonies from the selective blood tellurite plate of the primary inoculation to the Elek test, prior to isolation, purification and identification of the culture. Incubation time for the primary inoculation tellurite agar plates is 24–48 h and the optimized Elek test result is recorded after 24 h, thus, pathogenic corynebacteria in the clinical specimen could be detected within 48–72 h from the moment of sample plating. Early laboratory confirmation of diphtheria diagnosis allows timely initiation of specific antitoxin therapy, which may prevent the development of complications or even death of the patient, as well as to begin counter-epidemic measures as soon as possible. The cultures can then be submitted to a reference laboratory for identification, DT gene detection, confirmation of toxigenicity and molecular typing.

The accelerated diagnostic approach—application of suspicious colonies from the tellurite plate to the Elek test, was tested during the diphtheria epidemic in Russia in the 1990s [5]. There have also been some lessons learned from this epidemic that are worth recounting here. It is reasonable to study several colonies from the plate of primary inoculation in the Elek test, since both toxigenic and non-toxigenic strains can be present in a clinical specimen from one patient. To test as many colonies as possible on the primary inoculation plate, in addition to the isolated colonies, it is recommended that a mixture of 5–10 suspicious colonies be examined in the Elek test. In cases where it becomes necessary to study a large number of samples in the laboratory, up to 4 antitoxin discs can be placed in a Petri dish with a diameter of 10 cm. In the case of using a purified antitoxin and with sufficient personnel experience in setting the Elek test, it is even possible to place 3 positive controls and 3 test cultures around one disk. The negative control strain could be omitted and replaced by the test culture since the purified antitoxin does not produce non-specific precipitin lines in the Elek test. In this case, the toxigenicity of up to 12 test cultures can be simultaneously examined on one Petri dish [4, 5, 7]. To avoid misinterpretation, colonies from one patient should be inoculated around the same disc with antitoxin. When using a 10 cm Petri dish, the volume of agar should be 15 ml (12 ml of Elek medium and 3 ml of bovine serum).

Nevertheless, despite the optimization, the Elek test remains a rather laborious and time-consuming method. Therefore, a modern, simple, reliable and commercially available test is urgently needed, which can be used for the rapid (within 24 h) detection of toxigenic corynebacteria in any clinical laboratory in the absence of sophisticated equipment and specially trained personnel. Such a method, for example, can be the recently developed LFIA [10].
